# The rs2516839 Polymorphism of the *USF1* Gene May Modulate Serum Triglyceride Levels in Response to Cigarette Smoking

**DOI:** 10.3390/ijms160613203

**Published:** 2015-06-10

**Authors:** Pawel Niemiec, Tomasz Nowak, Tomasz Iwanicki, Sylwia Gorczynska-Kosiorz, Anna Balcerzyk, Jolanta Krauze, Wladyslaw Grzeszczak, Maria Wiecha, Iwona Zak

**Affiliations:** 1 School of Health Sciences in Katowice, Medical University of Silesia, Department of Biochemistry and Medical Genetics, Medykow Str 18, 40-752 Katowice, Poland; E-Mails: tn1984@o2.pl (T.N.); t_iwanicki@wp.pl (T.I.); izak@sum.edu.pl (I.Z.); 2 School of Medicine with the Division of Dentistry in Zabrze, Medical University of Silesia, Department of Internal Medicine, Diabetes and Nephrology, 3 Maja Str 13-18, 41-800 Zabrze, Poland; E-Mails: skosiorz@sum.edu.pl (S.G.-K.); abalcerzyk@sum.edu.pl (A.B.); wgrzeszczak@sum.edu.pl (W.G.); 3 School of Medicine in Katowice, Medical University of Silesia, 1st Department of Cardiac Surgery in Upper Silesian Center of Cardiology in Katowice, Ziolowa Str 47, 40-635 Katowice, Poland; E-Mail: jolakra@poczta.fm; 4 Regional Centre of Blood Donation and Blood Treatment in Raciborz, Sienkiewicza Str 3, 47-400 Raciborz, Poland; E-Mail: sekretariat@rckik.pl

**Keywords:** USF1, polymorphism, cigarette smoking, CAD, triglycerides, gene-traditional risk factors interactions

## Abstract

Single nucleotide polymorphisms (SNPs) of the *USF1* gene (upstream stimulatory factor 1) influence plasma lipid levels. This study aims to determine whether USF1 SNPs interact with traditional risk factors of atherosclerosis to increase coronary artery disease (CAD) risk. In the present study serum lipid levels and *USF1* gene polymorphisms (rs2516839 and rs3737787) were determined in 470 subjects: 235 patients with premature CAD and 235 controls. A trend of increasing triglycerides (TG) levels in relation to the C allele dose of rs2516839 SNP was observed. The synergistic effect of cigarette smoking and C allele carrier state on CAD risk was also found (SIM = 2.69, *p* = 0.015). TG levels differentiated significantly particular genotypes in smokers (1.53 mmol/L for TT, 1.80 mmol/L for CT and 2.27 mmol/L for CC subjects). In contrast, these differences were not observed in the non-smokers subgroup (1.57 mmol/L for TT, 1.46 mmol/L for CT and 1.49 mmol/L for CC subjects). In conclusion, the rs2516839 polymorphism may modulate serum triglyceride levels in response to cigarette smoking. Carriers of the C allele seem to be particularly at risk of CAD, when exposed to cigarette smoking.

## 1. Introduction

Lipid disorders are one of the most important predictors of atherosclerotic coronary artery disease (CAD). Polymorphic variants of key genes involved in lipid metabolism may also significantly influence the risk of CAD, modifying the system’s response. The *USF1* (upstream stimulatory factor 1) gene (1q23.3) is one of the regulatory genes of lipid metabolism [1]. Its locus was initially identified by linkage mapping studies and it was associated with familial combined hyperlipidemia [2,3,4]. The *USF1* gene encodes the USF1 polypeptide, a subunit of the dimeric transcription factor USF, which belongs to the DNA-binding proteins [5]. USF regulates expression of numerous genes involved in cellular proliferation, cell cycle, stress and immune response, as well as lipid and carbohydrate metabolism, including apolipoprotein E gene (*APOE*), ATP-binding cassette, sub-family A, member 1 gene (*ABCA1*), apolipoprotein A-V gene (*APOA5*), angiotensinogen gene (*AGT*) and many more [4,5]. Through the impact on target genes, USF is involved in many processes like the synthesis of fatty acids, β-oxidation, binding and transport of lipids in the bloodstream, and prostanoid metabolism.

In many studies, *USF1* gene polymorphisms have been associated either with familial combined hyperlipidemia or one of its component traits [2,4,6,7,8,9,10,11,12,13,14,15]. They were also analyzed in the context of diabetes mellitus type II, metabolic syndrome [8,9,10,16] and CAD [11,13,14]. However, there are significant differences in the observed results, especially those concerning the impact of the *USF1* gene polymorphisms on lipid levels and on the phenotype of the disease [6,17,18,19].

The presence of those substantial differences suggests that additional factors may affect the influence of *USF1* genotypes on lipid parameters. Therefore, in the current study we tried to identify the factors that may modify these effects. We analyzed two SNPs of the *USF1* gene, namely rs2516839 (c.161043331C>T) and rs3737787 (c.161039733C>T), and their possible associations with premature CAD. In addition to the classical case-control analysis, we also analyzed: (1) the impact of the *USF1* genotype variants on serum lipid parameters; (2) possible interactions of both *USF1* gene polymorphisms with traditional risk factors of atherosclerosis increasing the risk of CAD and (3) we searched for traditional risk factors influencing the differences in lipid parameters, dependent on *USF1* gene alleles.

## 2. Results

### 2.1. Characteristics of the Study Groups

Clinical and biochemical parameters of patients and controls are shown in Table 1. There were 73.2% cases who had suffered from myocardial infarction and 63.8% patients with critical stenoses (>90%) in coronary vessels. The vast majority of patients were treated with statins (97.9%). There is no information about statin therapy in blood donors. Due to inclusion criteria to this group, the use of statins seems to be doubtful. CAD patients showed increased levels of total cholesterol (TC), LDL cholesterol (LDL-C) and triglycerides (TG), and a higher body mass index (BMI). The level of HDL cholesterol (HDL-C) was significantly lower in CAD patients. Cigarette smokers, hypertensive subjects and diabetics were significantly more frequent in CAD group. The high value of the OR for hypertension resulted from the recommendations of Polish Centers of Blood Donation and Blood Treatment, which preclude blood sampling from donors with high blood pressure (see Experimental Section).

**Table 1:** Clinical and biochemical characteristics of the coronary artery disease patients and blood donors.

Characteristic	CAD (*n* = 235)	BD (*n* = 235)	OR (95% CI) Univariate Analysis	*p*
Age (years), mean ± SD	44.61 ± 5.90	43.97 ± 5.96	-	0.24
Male gender, % (No.)	70.2 (165)	70.2 (165)	1.00 (0.67–1.48)	1.00
BMI, mean ± SD	27.11 ± 4.21	26.23 ± 3.88	-	0.027
Cigarette Smoking, % (No.)	57.9 (136)	27.7 (65)	3.59 (2.44–5.28)	<10^−10^
Hypertension, % (No.)	56.6 (133)	2.6 (6)	49.77 (21.26–116.51)	<10^−10^
Diabetes mellitus, % (No.)	8.5 (20)	0 (0)	2.09 (1.90–2.31) *^a^*	<10^−6^
Familial history of CAD, % (No.)	35.3 (83)	0 (0)	2.54 (2.25–2.88) *^a^*	<10^−7^
TC, mmol/L, mean ± SD	5.74 ± 1.46	5.09 ± 1.22	-	<10^−7^
LDL-C, mmol/L, mean ± SD	4.01 ± 1.24	3.15 ± 1.18	-	<10^−10^
HDL-C, mmol/L, mean ± SD	1.09 ± 0.31	1.44 ± 0.56	-	<10^−10^
TG, mmol/L, mean ± SD	1.87 ± 0.98	1.40 ± 0.73	-	<10^−8^

^a^ Risk Ratio values (95% CI), univariate analysis; CAD—coronary artery disease patients; BD—blood donors; OR—Odds Ratio.

### 2.2. Case-Control Association Analysis of the USF1 Gene Polymorphisms and CAD

Genotype frequencies of both *USF1* polymorphisms were compatible with the Hardy-Weinberg equilibrium in both groups (for rs2516839: *p* = 0.81 in CAD group, *p* = 0.72 in controls; for rs3737787: *p* = 0.50 in CAD group, *p* = 0.92 in controls).

Data from genotyping are shown in Table 2. There were no statistically significant differences in the frequencies of genotypes and alleles of both polymorphisms between the groups. There were also no differences in male and female subgroups of CAD patients and controls (Supplementary Tables S1 and S2, respectively).

**Table 2:** The frequency of genotypes and alleles of the *USF1* gene polymorphisms in the groups of coronary artery disease (CAD) patients and blood donors.

Genotype, Allele	CAD (*n* = 235) % (*n*)	BD (*n* = 235) % (*n*)	OR (95% CI) Univariate Analysis		*p*
**rs2516839**					
CC	16.2 (38)	17.4 (41)	*vs.* TT + CT	0.91 (0.56–1.48)	0.71
CT	50.6 (119)	45.5 (107)	-	-	-
TT	33.2 (78)	37.0 (87)	*vs.* CC + CT	0.84 (0.58–1.23)	0.38
CC + CT	66.8 (157)	62.9 (148)	*vs.* TT	1.18 (0.81–1.73)	0.38
TT + CT	83.8 (197)	82.5 (194)	*vs.* CC	1.10 (0.68–1.78)	0.71
C	41.5 (195)	40.2 (189)	*vs.* T	1.05 (0.81–1.37)	0.69
T	58.5 (275)	59.8 (281)	*vs.* C	0.95 (0.73–1.23)	0.69
**rs3737787**					
CC	44.7 (105)	45.1 (106)	*vs.* TT + CT	0.98 (0.68–1.41)	0.93
CT	46.8 (110)	45.1 (106)	-	-	-
TT	8.5 (20)	9.8 (23)	*vs.* CC + CT	0.86 (0.46–1.61)	0.63
CC + CT	91.5 (215)	90.2 (212)	*vs.* TT	1.16 (0.62–2.19)	0.63
TT + CT	55.3 (130)	54.9 (129)	*vs.* CC	1.01 (0.71–1.46)	0.93
C	68.1 (320)	67.7 (318)	*vs.* T	1.02 (0.78–1.34)	0.89
T	31.9 (150)	32.3 (152)	*vs.* C	0.98 (0.75–1.29)	0.89

CAD—coronary artery disease patients; BD—blood donors; OR—Odds Ratio.

The presence of a haplotype block, size 3.6 kb, was shown, according to the Four Gamete Rule method. Linkage disequilibrium measures were: D′ = 0.93 and r^2^ = 0.29. The C_rs2516839_T_rs3737787_ haplotype was significantly more frequent in CAD group, even after the Bonferroni correction (Table 3), however, due to its low frequency, confirmation of these results would require significantly larger study groups.

**Table 3:** Association of the *USF1* gene haplotypes with coronary artery disease.

Haplotype *		CAD		BD		χ^2^	OR (95% CI) Univariate Analysis	*p*
		+ (%)	− (%)	+ (%)	− (%)			
H1	CT	1.2	98.8	0	100	5.18	2.04 (1.90–2.19) *^a^*	0.014
H2	TT	28.8	71.2	32.7	67.3	1.50	0.83 (0.62–1.12)	0.22
H3	CC	41.5	58.5	41.0	59.0	0.02	1.02 (0.77–1.34)	0.89
H4	TC	28.5	71.5	26.3	73.7	0.52	1.12 (0.82–1.52)	0.47

* First allele of rs2516839 SNP, second of rs3737787 SNP; *^a^* Risk Ratio value (95% CI), univariate analysis; CAD—coronary artery disease patients; BD—blood donors; OR—Odds Ratio; H—haplotype.

### 2.3. Influence of the USF1 Polymorphic Variants on Serum Lipid Levels

TC, HDL-C, LDL-C and TG levels were compared between particular genotypes of *USF1* polymorphisms. Only the levels of TG differed between the genotypes of the rs2516839 polymorphism. TG levels increased in relation to the C allele dose (Table 4). This trend was observed in the CAD group as well as in the entire group (CAD + BD subjects), however, disappeared after adjusting for Bonferroni correction. TC, HDL-C, LDL-C levels and BMI values did not differ between the rs2516839 polymorphism genotypes (Supplementary Table S3).

**Table 4:** Triglyceride (TG) serum levels in regard to genotypes of *USF1* gene, rs2516839 polymorphism.

Group	CC	CT	TT	CC + CT	CT + TT
	TG (mmol/L), Mean ± SD				
**CAD**	2.17 ± 1.38 *^a^*^,*b*^	1.86 ± 0.87	1.73 ± 0.88	1.94 ± 1.03	1.81 ± 0.88
**BD**	1.48 ± 0.80	1.36 ± 0.70	1.40 ± 0.74	1.39 ± 0.73	1.38 ± 0.71
**CAD + BD**	1.82 ± 1.17 *^c^*^,*d*^	1.62 ± 0.83	1.56 ± 0.82	1.67 ± 0.93	1.59 ± 0.83

*^a^* CC *vs.* CT + TT (CAD), *p* = 0.071, NS; *^b^* CC *vs.* TT (CAD), *p* = 0.039 *; *^c^* CC *vs.* CT + TT (CAD + BD), *p* = 0.043 *; *^d^* CC *vs.* TT (CAD + BD), *p* = 0.048 *; * lack of significance after Bonferroni correction (*p* ≥ 0.025); CAD—coronary artery disease patients; BD—blood donors; NS—not statistically significant before and after Bonferroni correction.

Lipid profiles and BMI values did not differ between the rs3737787 polymorphism genotypes (Supplementary Table S4).

### 2.4. USF1 Gene Polymorphisms-Traditional Risk Factors Interactions Increasing the Risk of CAD

The presence of interactions between respective genotypes and classical risk factors of CAD such as cigarette smoking, overweight/obesity, male gender and hypertension was checked. To determine possible interactions between *USF1* gene variants and traditional risk factors of CAD, the 4 × 2 table approach of biological interactions was used. Dyslipidemias were excluded from these analyses because of the impact of *USF1* gene on serum lipid levels. An additive effect of genetic and traditional risk factors on CAD risk was also tested using univariate and multiple logistic regression approach, after adjustment for age, gender and other traditional risk factors of the disease.

A synergistic effect of rs2516839 SNP and cigarette smoking was found (Table 5). Cigarette smokers carrying the C allele had an increased risk of CAD compared with TT homozygous cigarette smokers and non-smoking C allele carriers (Table 5). Estimated CAD risk was about 170% greater than that predicted by assuming multiplication of the effects (SIM = 2.69, 95% CI; 1.21–6.01). The differences were significant also after Bonferroni correction, (*p* = 0.015) and the power of test was 95%, with a 95% two-sided confidence level. Obtained results indicate that cigarette smoking may increase the risk of CAD especially in the C allele carriers of the rs2516839 polymorphism.

The presence of this interaction was confirmed in the logistic regression model. Cigarette smokers carrying the C allele of the *USF1* gene rs2516839 polymorphism were more frequent in the CAD group than in controls (42.1% *vs.* 15.7%, OR = 3.90, 95% CI; 2.52–6.03, *p* < 10^−10^). The power of the test was 99.8%, with a 99.9% two-sided confidence level. These differences were statistically significant also in multivariate model, after adjustment for age, gender and other possible confounders like BMI value, lipid abnormalities, hypertension, family history of CAD and diabetes mellitus (OR = 3.24, 95% CI; 2.03–5.20, *p* < 10^−9^).

Variants of both *USF1* gene polymorphisms did not modulate the risk of CAD in response to exposure to other traditional risk factors, both in logistic regression analysis, as well as 4 × 2 table approach (Supplementary Table S5).

**Table 5:** Synergistic effect between C allele carrier state of the *USF1* gene rs2516839 polymorphism and cigarette smoking exposure.

CC + CT	Cigarette Smoking	CAD (*n* = 235)	BD (*n* = 235)	OR (95% CI), *p*	OR	SIM
0	0	41	59	1	-	-
0	1	37	28	1.90 (1.01–3.58), 0.045	OR_01 *vs.* 00_	-
1	0	58	111	0.75 (0.45–1.25), 0.27	OR_10 *vs.* 00_	-
1	1	99	37	3.85 (2.22–6.67), 2.1 × 10^−6^	OR_11 *vs.* 00_	2.69 *^a^*

*^a^* SIM = 2.69 (95% CI; 1.21–6.01), *p* = 0.015; CAD—coronary artery disease patients; BD—blood donors; OR—Odds Ratio; OR_01 *vs.* 00_—OR for cigarette smoking exposure; OR_10 *vs.* 00_—OR for C allele carrier state; OR_11 *vs.* 00_—OR for co-exposure to genetic and traditional risk factor; SIM—multiplicative synergy index.

### 2.5. Genotype Specific Modulation of Serum Triglyceride Levels in Response to Cigarette Smoking

As we found the interaction between the C allele of the rs2516839 SNP and cigarette smoking, we decided to check whether the effect of the rs2516839 polymorphism on serum triglyceride levels is dependent on smoking status. For this purpose, serum TG concentrations were determined for each genotype of the rs2516839 polymorphism, in smokers and non-smokers from both studied groups (Table 6). TG levels increased with the number of copies of the C allele, but only in smokers. Homozygous CC cigarette smokers had the highest concentration of triglycerides, whereas TT homozygotes the lowest. Interestingly, these differences were not observed in the non-smokers subgroup (Table 6).

**Table 6:** Triglyceride (TG) serum levels in regard to genotypes of *USF1* rs2516839 polymorphism in smokers and non smokers subgroups.

Genotype	Smokers (*n* = 201)		Non-Smokers (*n* = 269)	
	*n*	TG (mmol/L) ± SD	*n*	TG (mmol/L) ± SD
CC	33	2.27 ± 1.48 *^a^*^,*b*,*c*,*d*^	46	1.49 ± 0.73
CT	103	1.80 ± 0.94 *^e^*^,*f*^	123	1.46 ± 0.70
TT	65	1.53 ± 0.84 *^g^*	100	1.57 ± 0.81
**Carrier State**				
CC + CT	136	1.92 ± 1.11 *^h^*^,*i*^	169	1.47 ± 0.71
CT + TT	168	1.70 ± 0.91	223	1.51 ± 0.75

*^a^ vs.* TT (smokers), *p* = 0.002; *^b^ vs.* CT (smokers), *p* = 0.028 *; *^c^ vs.* CT + TT (smokers), *p* = 0.004; *^d^ vs.* CC (non-smokers), *p* = 0.003; *^e^ vs.* TT (smokers), *p* = 0.052, NS; *^f^ vs.* CT (non-smokers), *p* = 0.002; *^g^ vs.* TT (non-smokers), *p* = 0.79, NS; *^h^ vs.* TT (smokers), *p* = 0.016; *^i^ vs.* CC + CT (non-smokers), *p* = 0.00003; * Lack of significance after Bonferroni correction (*p* ≥ 0.025); CAD—coronary artery disease patients; NS—not statistically significant before and after Bonferroni correction.

To confirm the results shown in Table 6, and take into account the influence of possible confounders, we conducted an analysis using multivariate logistic regression model, after adjustment for traditional risk factors of CAD (Table 7). The CC genotype was associated with increased TG levels in cigarette smokers (*p* = 0.008) and TT genotype was associated with decreased TG levels (*p* = 0.007). There was no association between CT genotype and TG levels in cigarette smokers (*p* = 0.96). In contrast, there was no association between TG levels and genotypes of the *USF1* gene rs2516839 polymorphism in non-smokers subgroup (Table 7). On the basis of the above data, it can be concluded that the rs2516839 polymorphism of the *USF1* gene may modulate serum triglyceride levels in response to cigarette smoking.

**Table 7:** The influence of genotype variants of the *USF1* gene rs2516839 polymorphism on triglyceride levels increase in subgroups of smokers and non-smokers.

Genotype	Smokers (*n* = 201)		Non-Smokers (*n* = 269)	
	OR (95% CI)	*p* *	OR (95% CI)	*p* *
CC	1.81 (1.16–2.82)	0.008	1.04 (0.47–2.29)	0.59
CT	0.99 (0.69–1.43)	0.96	0.83 (0.46–1.48)	0.52
TT	0.58 (0.32–0.86)	0.007	1.20 (0.66–2.16)	0.54
**Carrier State**				
CC + CT	1.72 (1.16–2.56)	0.007	0.83 (0.46–1.50)	0.54
CT + TT	0.64 (0.46–0.89)	0.007	0.96 (0.44–2.12)	0.92

* After adjustment for age, gender, BMI value, lipid abnormalities, hypertension, family history of CAD and diabetes mellitus.

## 3. Discussion

The present work is the first study analyzing polymorphisms of the *USF1* gene in the Polish population. A limitation of the present study is the fact that the analyses were performed in the group with a relatively small number of participants. But it has also its advantages, because of ethnical homogeneity of the subjects (only white polish Caucasians, inhabitants of Upper Silesia region were included). The frequencies of alleles of the rs2516839 in our study are similar to those observed in other Caucasian populations including German [9,10], Finnish [6,13], French-Canadian [14] and Australian of European origin [7]. The frequencies of alleles of the rs3737787 polymorphism also did not differ significantly from those observed previously in Caucasians [10,14,15].

There was no effect of analyzed polymorphisms on the risk of CAD in the present study. Because of the marginal frequency of the CA haplotype in our population, obtained results should be interpreted with great caution, however, rare haplotypes should not be ignored in the genetic studies of diseases with complex pathophysiology [20]. Case-control association studies concerning the role of *USF1* gene polymorphisms on CAD risk are not numerous. In the study of Komulainen *et al.* [13] the effect of *USF1* polymorphisms on CAD risk and overall mortality was showed, but only in females. The effect concerned allelic variants of rs2073658 polymorphism, but not rs2516839 [13]. In a study on U.S. whites, the frequencies of genotypes and alleles of rs3737787 SNP did not differ between CAD and non-CAD subjects [11]. There are also few functional studies concerning the influence of *USF1* polymorphisms on atherosclerotic phenotype. In the autopsy series of 700 Finnish middle-aged men, *USF1* polymorphisms were analyzed with quantitative morphometric measurements of coronary atherosclerosis [17]. The TT and CT genotypes of rs2516839 were associated with the proportion of advanced atherosclerotic plaques, area of calcification of coronary arteries and an increased risk of sudden cardiac death [17]. Contrary to these results, in the study of Fan *et al.* conducted on younger Finnish population, the C allele was associated with a higher mean carotid intima-media thickness, but only in females [6]. These results are supported by the study carried out on the Chinese population of atherosclerotic stroke patients [19], where the T allele carriers of rs2516839 SNP had a lower total unstable carotid plaque area than the CC homozygotes.

In the present work, the levels of triglycerides differed between the rs2516839 genotypes. A trend of increasing TG levels in relation to the C allele dose was observed, however, its statistical significance disappeared after the correction for multiple testing. Our findings are in agreement with previous studies, where C allele was associated with increased lipid levels [6,13,14]. Although some studies imply that the T allele is rather related to the presence of lipid abnormalities [7,8] whereas other show no effect of rs2516839 SNP on lipid levels [15,19]. Similar differences concern the rs3737787 polymorphism [8,10,11,12,18]. The presence of these discrepancies results from the fact that additional factors may modulate the functioning of both the *USF1* gene and the phenotype dependent on *USF1* polymorphisms. For the time being, sex-related allelic differences [6,13] and *USF1*-other genes interactions have been documented [7,9].

In our study, a synergistic effect of cigarette smoking and rs2516839 on CAD risk was found. Cigarette smoking increased the risk of CAD especially in the C allele carriers. Our results also indicated that TG levels differentiated particular genotypes but only in smokers, independently of other traditional risk factors.

The molecular mechanism of interaction between the *USF1* gene and cigarette smoking remains unknown, but it may be partly explained by the fact that USF1 is considered as a stress-responsive transcription factor. This issue was comprehensively discussed in the review of Corre and Galibert [5], summarizing that USF1 has antiproliferative properties and regulates many genes of cell cycle and tumor suppression (*i.e.*, cyclin B1 gene—*CCNB1*, cyclin-dependent kinase 1 gene—*CDK1*, breast cancer 2 gene—*BRCA2*, tumor protein p53 gene—*TP53*, adenomatous polyposis coli gene—*APC* and others). Accordingly, a loss of transcriptional activity of *USF1* in many cancer cell lines was observed (discussed in Corre and Galibert [5]). Interestingly, according to Fan *et al.* study [6], the *USF1* expression was significantly lower also in atherosclerotic plaque specimens compared with the control tissue [6], and CC homozygotes of rs2516839 SNP had a lower expression of the *USF1* gene in relation to the T allele carriers. In this context, reduced expression of *USF1* in C allele carriers may impair the cellular response to cigarette smoking, and increase the predisposition to atherosclerotic CAD. The question whether the USF transcription factor is involved in the cellular response to smoking (nicotine or any other component of tobacco smoke) remains open, however, it was demonstrated that nicotine enhanced the USF1 translocation from the cytoplasm to the nucleus in odontoblast cells [21].

There is, however, strong evidence for links between cigarette smoking and phenotype partly dependent on *USF1* gene polymorphisms [22,23]. It is well documented that cigarette smoking influences lipid concentrations. Results of a large meta-analysis of Craig *et al.* [22] indicated that cigarette smokers had significantly higher serum levels of TC (3.0%), TG (9.1%), VLDL-C (10.4%) and LDL-C (1.7%), and lower serum levels of HDL-C (−5.7%) compared with non-smokers, and the effect was dose-related. Cigarette smoking also influences lipid metabolism. The activity of lipoprotein lipase in skeletal muscles is reduced and this in turn decreases lipid clearance. Lipoprotein lipase activity is insulin-dependent and insulin resistance among smokers was observed in many studies [24,25].

In conclusion, C allele carriers of rs2516839 SNP seem to be particularly at risk of CAD, when exposed to cigarette smoking. Cigarette smoking influences TG levels, which increase with the number of copies of the C allele. The existence of numerous positive feedbacks between the *USF1* gene, targets of USF transcription factor, cigarette smoking and lipid abnormalities may intensify the effects associated with each of the factors analyzed individually. In order to understand the relationship between *USF1* gene polymorphisms, cigarette smoking and lipid levels on atherosclerotic phenotype, further functional studies are necessary.

## 4. Experimental Section

### 4.1. Subjects

Four hundred and seventy Polish Caucasians, inhabitants of Upper Silesia were studied. The first group (CAD) consisted of 235 patients with angiographically proven premature CAD (70 females and 165 males), aged 27–55 years (mean 44.61 ± 5.90). Group 2 (BD) included 235 blood donors matched by age and gender, with no signs of CAD and with negative familial history of CAD as inclusion criteria. CAD subjects were selected from patients admitted to the: (1) 1st Department and Clinic of Cardiology at the Upper Silesian Center of Cardiology in Katowice; (2) 1st Department of Cardiac Surgery at the Upper Silesian Center of Cardiology in Katowice. They were classified for the study by the same cardiologist. Controls were recruited from the Regional Centers of Blood Donation and Blood Treatment in Katowice and Raciborz. Following recommendations of the Polish Centers of Blood Donation and Blood Treatment, the blood samples were obtained only from subjects with systolic blood pressure <140 and diastolic blood pressure <90 on the day of blood collection. Inclusion and exclusion criteria, details of the medical interview, diagnosis and evaluation as well as criteria for CAD, myocardial infarction and traditional risk factors were described previously [26]. The study protocol was approved by the Ethics Committee of the Medical University of Silesia in Katowice, Poland. All subjects gave written informed consents. The methods used in this study were in accordance with the Helsinki Declaration of 1975 and its further revisions.

### 4.2. Serum Lipids Measurement

Total serum cholesterol, HDL cholesterol and triglycerides were measured by enzymatic colorimetric methods (Analco, Warsaw, Poland). LDL cholesterol levels were calculated according to the Friedewald formula [27] in subjects with triglycerides levels below 4.4 mmol/L.

### 4.3. DNA Extraction and Genotyping

Genomic DNA was extracted from peripheral leukocytes using the MasterPure genomic DNA purification kit (Epicentre Technologies, Madison, WI, USA). The *USF1* gene polymorphisms were genotyped using the TaqMan^®^ Pre-designed SNP Genotyping Assay (Applied Biosystems, Foster City, CA, USA). The total volume of 20 µL reaction mix included: 10 µL of TaqMan^®^ Genotyping Master Mix (Cat. # 4371355), 1 µL of probe (TaqMan® Pre-designed SNP Genotyping Assay, Cat. # 4351376: ID; C_1839183_10 for rs2516839 or ID; C_1459759_20 for rs3737787 polymorphism), 1 µL of a DNA template (15 ng/µL) and 8 µL of deionized water. The probe was diluted with the TE buffer (1:1) before the reaction. Polymerase chain reaction amplification was performed according to the manufacturer’s specifications. Genotyping was performed using the 7300 Real-Time PCR System (Applied Biosystems). Correctness of genotyping of both polymorphisms was checked by re-genotyping 10%–15% of the samples. Repeatability of results was 100%.

### 4.4. Statistical Analysis

Data were analyzed using the Statistica 10.0 software (STATSOFT, Tulsa, OK, USA). Normality of distribution was assessed by the Shapiro-Wilk test and then a comparison of quantitative data was performed by the Mann-Whitney *U* test (for variables with non-normal distribution) or the student’s *t* test (for variables with normal distribution). The Hardy-Weinberg equilibrium was tested in all groups by a χ^2^ test as well as comparisons of genotypes and alleles frequencies between cases and controls. When the number of subjects in the subgroup was lower than 10 the Fisher’s correction was used. Odds ratios (OR) as well as their 95% confidence intervals (CI) were computed using an univariate analysis. Multiple logistic regression analysis was used after adjustment for age, sex and traditional risk factors of CAD (cigarette smoking, lipid levels, BMI value, hypertension, family history of CAD and diabetes mellitus). Risk ratio values (95% CI) were used when the number of individuals in any of the analyzed subgroups was 0. The effective sample size and statistical power of association analyzes were computed using *Epi Info*™ *7.1.1.0* developed by Centers for Disease Control and Prevention (Atlanta, GA, USA).

Haplotype blocks were defined by the HaploView software [28] using the Four Gamete Rule method. D′ and r^2^ values were used as linkage disequilibrium measures. A haplotype phase was determined on the basis of the population genetic data using the modified PHASE algorithm [29] used in the SNPator script [30].

To determine possible interactions between *USF1* genotypes and traditional risk factors of CAD, the 4 × 2 table approach was used. OR values obtained from 4 × 2 table comparisons were used for calculating multiplicative synergy indices (SIMs). SIM is a ratio of the observed effect with the joint exposure to genetic and traditional factors (OR_11_) divided by the effect predicted for the joint exposure assuming multiplication of the effects observed in the presence of either a traditional (OR_01_) or genetic factor (OR_10_). This method was used only in the case when the number of subjects from each compared group (not exposed, exposed only to genetic factor, exposed only to traditional risk factor or exposed to both factors) was >0. The following formula of SIM was used [31]:

SIM = OR_11_/OR_01_∙OR_10_

Confidence intervals (at a 95% confidence level) for SIMs were calculated using the script described by Cortina-Borja *et al.* [32].

In univariate analyses statistical significance was accepted at *p* < 0.05. The *p* values were adjusted for multiple comparisons using the Bonferroni correction in the case of analyses of gene-traditional risk factors interactions and the effects of genotypes on serum lipid levels. Because each adjustment procedure included one SNP and one tested factor, the significance level was reduced to *p* < 0.025.

Because the statistics of biological interactions preclude the use of quantitative variables, an effect of *USF1* gene variants and risk factors on CAD risk and lipid levels was tested using univariate and multiple logistic regression approach, after adjustment for age, gender and traditional risk factors of CAD.

## Supplementary Materials

Supplementary materials can be found at http://www.mdpi.com/1422-0067/16/06/13203/s1.
