# Supplementary material for: The rs2516839 Polymorphism of the USF1 Gene May Modulate Serum Triglyceride Levels in Response to Cigarette Smoking
Source: Int J Mol Sci. 2015 Jun 10;16(6):13203–16. doi: 10.3390/ijms160613203 (PMC4490492; doi:10.3390/ijms160613203)
Supplement: Supplementary file 1 [file ijms-16-13203-s001.pdf]

Supplementary Information

Table S1. The frequency of genotypes and alleles of the *USF1* gene polymorphisms in the male subgroups of CAD patients and blood donors.

Genotype, Allele	CAD (*n* = 165) % (*n*)	BD (*n* = 165) % (*n*)	OR (95% CI) Univariate Analysis		*p*
rs2516839					
CC	18.2 (30)	19.4 (32)	*vs.* TT + CT	0.92 (0.53−1.61)	0.78
CT	46.7 (77)	45.5 (75)	-	-	-
TT	35.1 (58)	35.1 (58)	*vs.* CC + CT	1.00 (0.64−1.57)	1.00
CC + CT	64.9 (107)	64.9 (107)	*vs.* TT	1.00 (0.64−1.57)	1.00
TT + CT	81.8 (135)	80.6 (133)	*vs.* CC	1.08 (0.62−1.88)	0.78
C	41.5 (137)	42.1 (139)	*vs.* T	0.98 (0.72−1.33)	0.87
T	58.5 (193)	57.9 (191)	*vs.* C	1.03 (0.75−1.40)	0.87
rs3737787					
CC	46.7 (77)	46.1 (76)	*vs.* TT + CT	1.02 (0.66−1.58)	0.91
CT	42.4 (70)	44.8 (74)	-	-	-
TT	10.9 (18)	9.1 (15)	*vs.* CC + CT	1.22 (0.59−2.52)	0.58
CC + CT	89.1 (147)	90.9 (150)	*vs.* TT	0.82 (0.40−1.68)	0.58
TT + CT	53.3 (88)	53.9 (89)	*vs.* CC	0.98 (0.63−1.50)	0.91
C	67.9 (224)	68.5 (226)	*vs.* T	0.97 (0.70−1.35)	0.87
T	32.1 (106)	31.5 (104)	*vs.* C	1.03 (0.74−1.43)	0.87

CAD—coronary artery disease patients; BD—blood donors; OR—Odds Ratio.

Table S2. The frequency of genotypes and alleles of the *USF1* gene polymorphisms in the female subgroups of CAD patients and blood donors.

Genotype, Allele	CAD (*n* = 70) % (*n*)	BD (*n* = 70) % (*n*)	OR (95% CI) Univariate Analysis		*p*
rs2516839					
CC	11.4 (8)	12.9 (9)	*vs.* TT + CT	0.87 (0.32−2.41)	0.80
CT	60.0 (42)	45.7 (32)	-	-	-
TT	28.6 (20)	41.4 (29)	*vs.* CC + CT	0.57 (0.28−1.14)	0.11
CC + CT	71.7 (50)	58.6 (41)	*vs.* TT	1.77 (0.88−3.57)	0.11
TT + CT	88.6 (62)	87.1 (61)	*vs.* CC	1.14 (0.41−3.16)	0.80
C	41.4 (58)	35.7 (50)	*vs.* T	1.27 (0.79−2.06)	0.33
T	58.6 (82)	64.3 (90)	*vs.* C	0.78 (0.48−1.27)	0.33
rs3737787					
CC	40.0 (28)	42.9 (30)	*vs.* TT + CT	0.89 (0.45–1.74)	0.73
CT	57.1 (40)	45.7 (32)	-	-	-
TT	2.9 (2)	11.4 (8)	*vs.* CC + CT	0.23 (0.02–1.21)	0.10
CC + CT	97.1 (68)	88.6 (62)	*vs.* TT	4.39 (0.82–43.54)	0.10
TT + CT	60.0 (42)	57.1 (40)	*vs.* CC	1.12 (0.57–2.20)	0.73
C	68.6 (96)	65.7 (92)	*vs.* T	1.13 (0.69–1.88)	0.61
T	31.4 (44)	34.3 (48)	*vs.* C	0.87 (0.53–1.45)	0.61

CAD—coronary artery disease patients; BD—blood donors; OR—Odds Ratio.

Table S3. Serum levels of total cholesterol (TC), HDL cholesterol (HDL-C) and LDL cholesterol (LDL-C) and BMI in regard to genotypes of the *USF1* gene rs2516839 polymorphism.

Group	CC	CT	TT	CC + CT	CT + TT
			**TC (mmol/L), Mean ± SD**		
CAD	5.87 ± 1.32	5.66 ± 1.50	5.82 ± 1.48	5.71 ± 1.46	5.72 ± 1.49
BD	5.05 ± 1.10	5.07 ± 1.32	5.12 ± 1.15	5.07 ± 1.26	5.10 ± 1.24
CAD + BD	5.44 ± 1.27	5.38 ± 1.45	5.45 ± 1.36	5.40 ± 1.40	5.41 ± 1.41
			**HDL-C (mmol/L), Mean ± SD**		
CAD	1.07 ± 0.23	1.06 ± 0.32	1.12 ± 0.31	1.07 ± 0.30	1.09 ± 0.32
BD	1.45 ± 0.60	1.42 ± 0.55	1.47 ± 0.56	1.43 ± 0.56	1.44 ± 0.55
CAD + BD	1.26 ± 0.49	1.23 ± 0.48	1.31 ± 0.49	1.24 ± 0.48	1.27 ± 0.48
			**LDL-C (mmol/L), Mean ± SD**		
CAD	4.03 ± 1.14	3.99 ± 1.26	4.02 ± 1.27	4.00 ± 1.23	4.00 ± 1.26
BD	3.08 ± 1.08	3.17 ± 1.29	3.16 ± 1.08	3.14 ± 1.23	3.16 ± 1.20
CAD + BD	3.53 ± 1.20	3.59 ± 1.34	3.56 ± 1.24	3.57 ± 1.30	3.58 ± 1.30
			**BMI (kg/m^2), Mean ± SD**		
CAD	27.01 ± 3.24	27.36 ± 4.60	26.76 ± 3.99	27.28 ± 4.31	27.13 ± 4.73
BD	26.64 ± 3.40	26.04 ± 3.50	26.27 ± 4.51	26.21 ± 3.48	26.14 ± 3.98
CAD + BD	26.81 ± 3.31	26.71 ± 4.14	26.48 ± 4.28	26.74 ± 3.94	26.62 ± 4.20

Table S4. Serum levels of total cholesterol (TC), HDL cholesterol (HDL-C) and LDL cholesterol (LDL-C) and BMI in regard to genotypes of the *USF1* gene rs3737787 polymorphism.

Group	CC	CT	TT	CC + CT	CT + TT
			**TC (mmol/L), Mean ± SD**		
CAD	5.71 ± 1.34	5.78 ± 1.64	5.72 ± 1.10	5.75 ± 1.50	5.78 ± 1.56
BD	5.03 ± 1.27	5.12 ± 1.18	5.22 ± 1.17	5.07 ± 1.22	5.14 ± 1.18
CAD + BD	5.37 ± 1.35	5.45 ± 1.47	5.46 ± 1.15	5.41 ± 1.41	5.46 ± 1.42
			**HDL-C (mmol/L), Mean ± SD**		
CAD	1.10 ± 0.32	1.07 ± 0.29	1.08 ± 0.31	1.09 ± 0.31	1.07 ± 0.29
BD	1.43 ± 0.60	1.43 ± 0.48	1.54 ± 0.68	1.43 ± 0.54	1.45 ± 0.52
CAD + BD	1.27 ± 0.51	1.25 ± 0.44	1.32 ± 0.58	1.26 ± 0.48	1.26 ± 0.46
			**LDL-C (mmol/L), Mean ± SD**		
CAD	3.96 ± 1.21	4.06 ± 1.32	3.91 ± 0.98	4.01 ± 1.26	4.04 ± 1.27
BD	3.09 ± 1.23	3.21 ± 1.14	3.12 ± 1.14	3.15 ± 1.18	3.20 ± 1.14
CAD + BD	3.51 ± 1.29	3.64 ± 1.30	3.48 ± 1.14	3.58 ± 1.30	3.61 ± 1.27
			**TG (mmol/L), Mean ± SD**		
CAD	1.84 ± 1.06	1.90 ± 0.97	1.82 ± 0.70	1.87 ± 1.01	1.89 ± 0.93
BD	1.44 ± 0.78	1.35 ± 0.66	1.44 ± 0.79	1.39 ± 0.72	1.36 ± 0.68
CAD + BD	1.63 ± 0.94	1.63 ± 0.87	1.61 ± 0.76	1.63 ± 0.91	1.63 ± 0.85
			**BMI (kg/m^2), Mean ± SD**		
CAD	27.21 ± 4.26	26.97 ± 4.36	27.39 ± 2.91	27.08 ± 4.30	27.03 ± 4.17
BD	26.53 ± 3.64	25.91 ± 3.91	26.32 ± 4.83	26.22 ± 3.78	25.98 ± 4.07
CAD + BD	26.86 ± 3.95	26.42 ± 4.16	26.78 ± 4.10	26.64 ± 4.06	26.48 ± 4.14

Table S5. 4 × 2 Tables for distribution of C allele carrier state of the *USF1* gene rs2516839 polymorphism in relation to overweight/obesity (BMI ≥ 25) and male gender.

CC + CT	BMI ≥ 25	CAD (n = 235)	BD (n = 235)	OR (95% CI), p	OR	SIM
0	0	37	44	1	-	-
0	1	41	43	1.13 (0.62−2.09), 0.69	$OR_{01\ vs.\ 00}$	-
1	0	58	67	1.02 (0.58−1.80), 0.92	$OR_{10\ vs.\ 00}$	-
1	1	99	81	1.45 (0.86−2.46), 0.16	$OR_{11\ vs.\ 00}$	0.80 [a]
**CC + CT**	**Male Gender**	**CAD (n = 235)**	**BD (n = 235)**	**OR (95% CI), p**	**OR**	**SIM**
0	0	20	29	1	−	
0	1	58	58	1.45 (0.74−2.85), 0.45	$OR_{01\ vs.\ 00}$	
1	0	50	41	2.01 (1.00−4.05), 0.047	$OR_{10\ vs.\ 00}$	
1	1	107	107	1.45 (0.77−2.72), 0.25	$OR_{11\ vs.\ 00}$	1.76 [b]

[a] SIM = 0.80 (95% CI; 0.37–1.72), p = 0.57; [b] SIM = 1.76 (95% CI; 0.77–4.08), p = 0.18; CAD—coronary artery disease patients; BD—blood donors; OR—Odds Ratio; $OR_{01\ vs.\ 00}$—OR for cigarette smoking exposure; $OR_{10\ vs.\ 00}$—OR for C allele carrier state; $OR_{11\ vs.\ 00}$—OR for co-exposure to genetic and traditional risk factor; SIM—multiplicative synergy index.
